# Supplementary material for: Isolation of Small Extracellular Vesicles from Human Sera
Source: Int J Mol Sci. 2021 Apr 28;22(9):4653. doi: 10.3390/ijms22094653 (PMC8124960; doi:10.3390/ijms22094653)
Supplement: Supplementary file 1 [file ijms-22-04653-s001.zip › ijms-1189046-supplementary for conversion.pdf]

International Journal of *Molecular Sciences*

MDPI

Supplementary Materials

Isolation of Small Extracellular Vesicles from Human Sera

Małgorzata S. Małys [1], Christof Aigner [1,2], Stefan M. Schulz [1], Helga Schachner [1], Andrew J. Rees [1], Renate Kain [1,*]

1 Department of Pathology, Medical University Vienna, 1090 Vienna, Austria; malgorzata.malys@meduniwien.ac.at (M.S.M.); christof.aigner@meduniwien.ac.at (C.A.); stefan.schulz@meduniwien.ac.at (S.M.S.); helga.schachner@meduniwien.ac.at (H.S.); andrew.rees@meduniwien.ac.at (A.J.R.)

2 Division of Nephrology and Dialysis, Department of Medicine III, Medical University Vienna, 1090 Vienna, Austria

* Correspondence: renate.kain@meduniwien.ac.at

Supplementary Table S1. Small extracellular vesicles (sEV) properties obtained by nanoparticle tracking analysis presented for individual sEV preparations that were purified from the same serum sample by differential ultracentrifugation (DUC) and Exo-spin™. Additional letters (ie. b) next in donor column indicate additional timepoints of sampling.

Donor	Method	Mean Size (nm)	Median Size (nm)	Total Isolated Part. No ($\times 10^8$)	Part. No ($\times 10^8$)/mL Serum	Absolute No. of Part. <150 nm ($\times 10^8$)/mL Serum
D1	DUC	152.00	140.00	8.07	4.03	2.29
D2	DUC	138.67	137.67	1.21	0.61	0.35
D3	DUC	141.67	132.33	1.17	0.58	0.37
D4	DUC	149.33	138.33	1.13	0.57	0.32
D5	DUC	127.00	112.40	0.51	0.26	0.18
D6	DUC	143.00	137.33	0.56	0.28	0.16
D6b	DUC	136.67	126.67	2.40	1.20	0.82
D1b	DUC	174.00	160.00	1.11	0.55	0.24
D2b	DUC	170.67	157.67	1.68	0.84	0.37
D1	Exo-spin™	136.00	129.00	9.33	93.33	61.07
D2	Exo-spin™	150.67	145.33	0.63	6.27	3.53
D3	Exo-spin™	138.67	133.00	9.47	94.67	61.91
D4	Exo-spin™	133.67	126.00	24.00	240.00	162.56
D5	Exo-spin™	132.00	123.00	0.51	5.07	3.52
D6	Exo-spin™	136.00	126.67	33.47	334.67	223.67
D6b	Exo-spin™	102.33	95.30	254.67	2546.67	2284.36
D1b	Exo-spin™	132.33	113.67	90.67	906.67	658.54
D2b	Exo-spin™	131.00	116.67	25.07	250.67	180.48

Supplementary Table S2. Median APC fluorescence intensities of small extracellular vesicles preparations that were purified by differential ultracentrifugation (DUC) or Exo-spin™ and analysed by bead-based flow cytometry (MACSPlex). The data is shown as a heatmap with corresponding values included in the frame. Graded colour scale: low values (green) to high values (red).

Method	DUC						Exo-spin™					
Donor	D1	D2	D3	D4	D5	D6	D1	D2	D3	D4	D5	D6
CD3	6020	−119	2346	178	3082	2237	−70	−73	−47	−124	1384	−873
CD4	111	78	272	170	242	−39	−43	22	−67	321	1701	−12

**CD19**	−52	−13	145	−95	51	−129	9	54	−7	174	1755	125
**CD8**	533	555	604	1134	1836	−70	915	898	−84	516	3814	251
**HLA-DRDPDQ**	12308	3145	8943	13230	29040	5748	1667	676	753	1079	7604	1455
**CD56**	8444	95	1633	−405	5060	2985	−107	−125	−46	−101	1979	−681
**CD105**	2294	365	6116	1829	5061	1019	182	94	−779	721	2691	161
**CD2**	248	8	762	−152	84	−441	−22	−24	11	523	1399	−296
**CD1c**	−23	−209	48	197	−531	−667	−65	20	48	321	1066	−427
**CD25**	2312	456	−74	−338	2901	1466	−2	53	−257	159	1593	−223
**CD49e**	934	−133	1299	934	120	−358	−172	−94	77	343	1200	−424
**ROR1**	236	−92	700	420	481	−2	26	68	338	539	2149	215
**CD209**	30	−89	371	110	0	−208	−48	−10	197	544	1930	91
**CD9**	54533	9551	21281	15284	45462	27984	9180	1264	1433	2077	13367	3846
**SSEA-4**	249	−181	611	−593	84	−301	−271	−319	−536	149	1400	−397
**HLA-ABC**	42352	8409	5332	4058	39848	11074	1546	291	447	635	5506	681
**CD63**	43448	6166	27300	25949	41793	27304	25267	3040	5759	5696	21731	6354
**CD40**	6766	487	1570	711	5313	1708	269	−94	214	251	1838	227
**CD62P**	72653	6877	53538	17957	56853	43075	10402	844	6472	5054	10102	2157
**CD11c**	−114	−145	805	429	−452	−522	−94	−4	−11	477	1252	−225
**CD81**	3821	2048	7429	5620	13200	3313	4186	2133	912	1092	11397	2863
**MCSP**	−48	−57	378	115	0	−59	−141	−69	−8	80	1654	29
**CD146**	627	218	1088	2452	2773	244	164	60	222	441	1936	186
**CD41b**	80221	10709	34673	20092	60168	27171	18368	1217	5695	4717	17789	3779
**CD42a**	89024	7806	29923	19305	56520	26983	7922	819	1534	4072	8643	2627
**CD24**	29251	2760	8031	836	15203	5068	315	331	203	846	3251	795
**CD86**	760	144	1323	903	1354	245	91	128	−2	363	2105	201
**CD44**	1009	1032	1690	2280	2141	979	85	137	148	221	2551	10
**CD326**	248	9	1202	93	663	−3	−78	−9	151	569	1574	−48
**CD133/1**	201	−2	140	267	243	85	23	52	−146	687	1539	113
**CD29**	55982	7216	23514	17969	39334	15999	6205	724	914	3036	6224	1428
**CD69**	3409	440	2020	1186	2457	814	266	53	378	430	1747	18
**CD142**	1691	−26	2188	774	935	506	−76	−62	373	301	1770	194
**CD45**	4882	561	4110	256	2742	−13	45	284	−63	346	1302	−767
**CD31**	12166	614	1924	763	8290	2405	559	79	−52	488	2331	229
**REA ctrl**	−96	-23	331	829	−74	−202	−83	−21	635	731	1761	−2
**CD20**	110	−20	355	542	−37	−158	−59	10	168	251	1496	74
**CD14**	682	230	2636	−308	597	236	−26	165	−365	−918	1572	20
**mIgG1 ctrl**	81	−14	688	482	27	−4	22	67	−253	34	1641	173

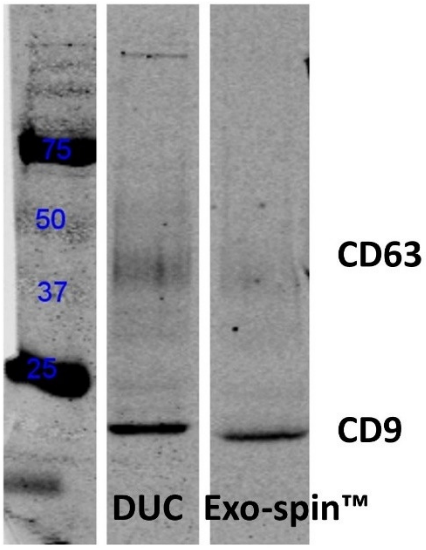

(a)

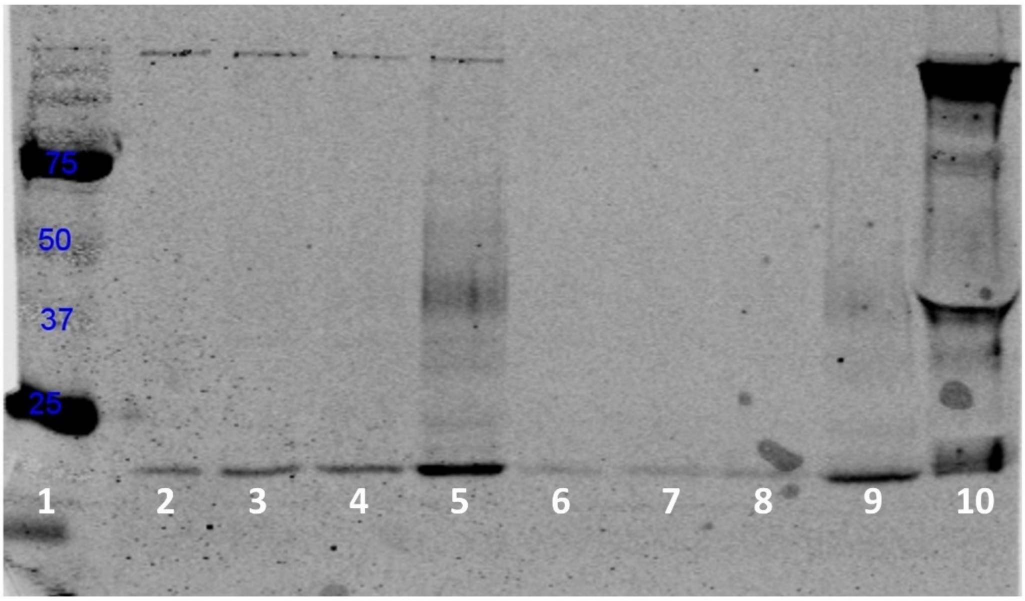

(b)

Supplementary Figure S1. Western blot of small extracellular vesicle (sEV) preparation purified by differential ultracentrifugation (DUC) and Exo-spin™ stained for CD9 and CD63 antibody. Image (**a**) presents the cut-out examples of sEV preparations purified from paired serum by DUC and Exo-spin05™. Image (**b**) shows the whole blot from which the lanes were cut out. Each lane stands for following: 1: marker; 2: sEV purified from plasma by DUC; 3: sEV purified from PPP by DUC; 4: sEV purified from defibrinated plasma by DUC; 5. sEV purified from serum by DUC; 6: sEV purified from plasma by Exo-spin05™; 7: sEV purified from PPP by Exo-spin05™; 8: sEV purified from defibrinated plasma by Exo-spin05™; 9: sEV purified from serum by Exo-spin05™; 10: sEV purified from serum by Exo-spin03™.

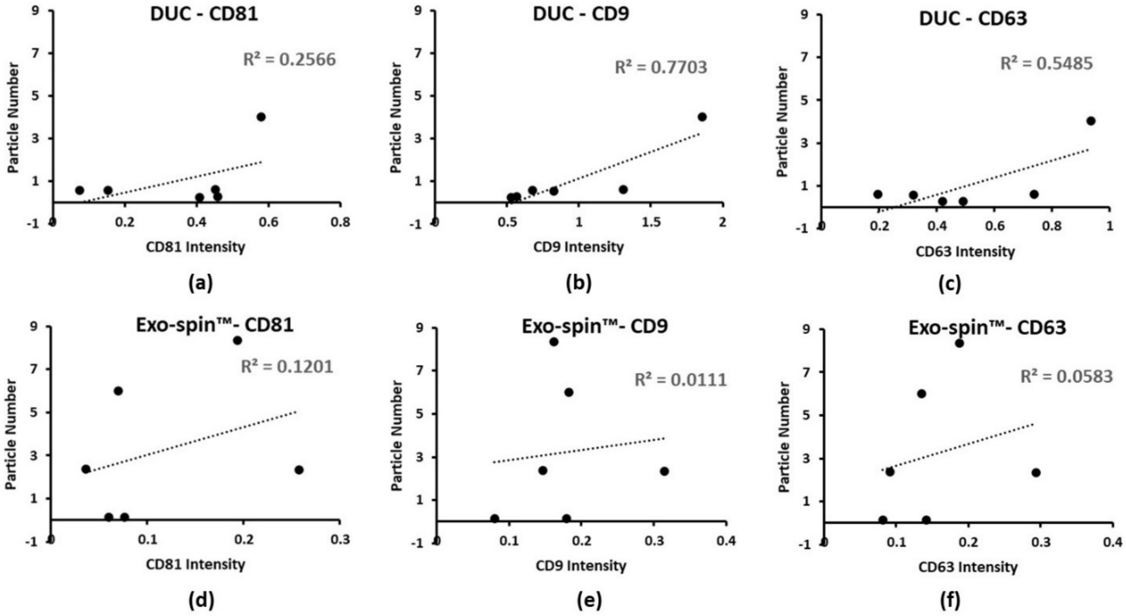

Supplementary Figure S2. Correlation between isolated particle numbers measured by nanoparticle tracking analysis and the mean intensity of marker expression measured by ELISA for small extracellular vesicles (sEV) purified by differential ultracentrifugation (DUC) and Exo-spin™ (Exo-spin05). Upper row (a-c): Differential ultracentrifugation; lower row (d-e): Exo-spin™. First column presents CD81, Second: CD9 and the third: CD63.

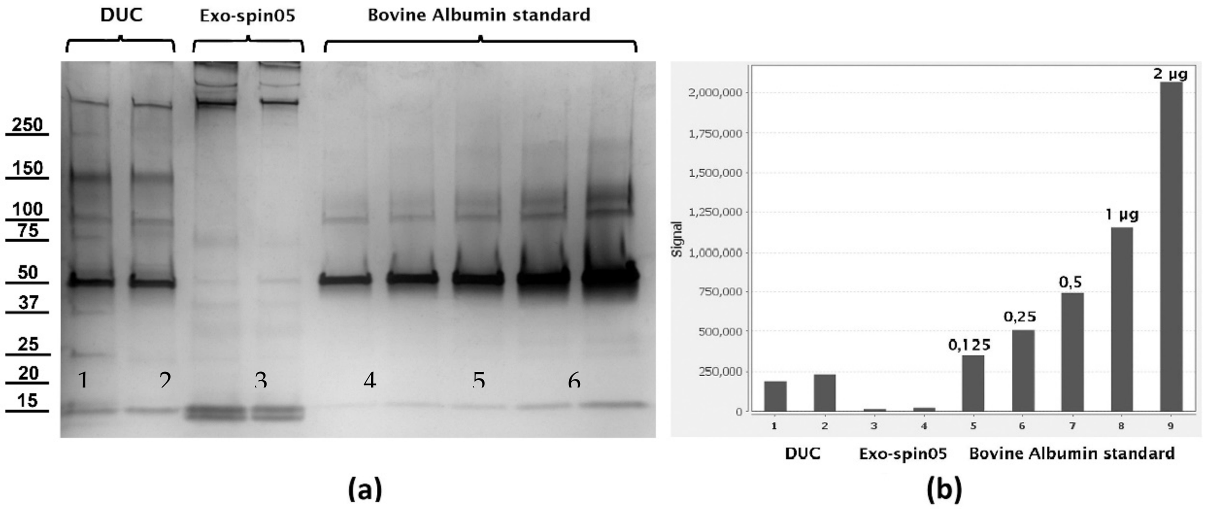

Supplementary Figure S3. Silver protein staining (**a**) of lysed small extracellular vesicles (lanes 1–4) purified from: serum (lane 1 and 3) and plasma (lane 2 and 4), respectively, isolated by differential ultracentrifugation (DUC) (lane 1 and 2) and Exo-spin™ (lane 3 and 4). Lanes 5–9 correspond to increasing amounts of Bovine Albumin Standard (0,125 µg (lane 5); 0,25 µg (lane 6); 0,5 µg (lane 7); 1 µg (lane 8); 2 µg (lane 9)). Figure (**b**) displays the measured signal intensities of each band in the area of molecular weight corresponding to the albumin (66 kDa).

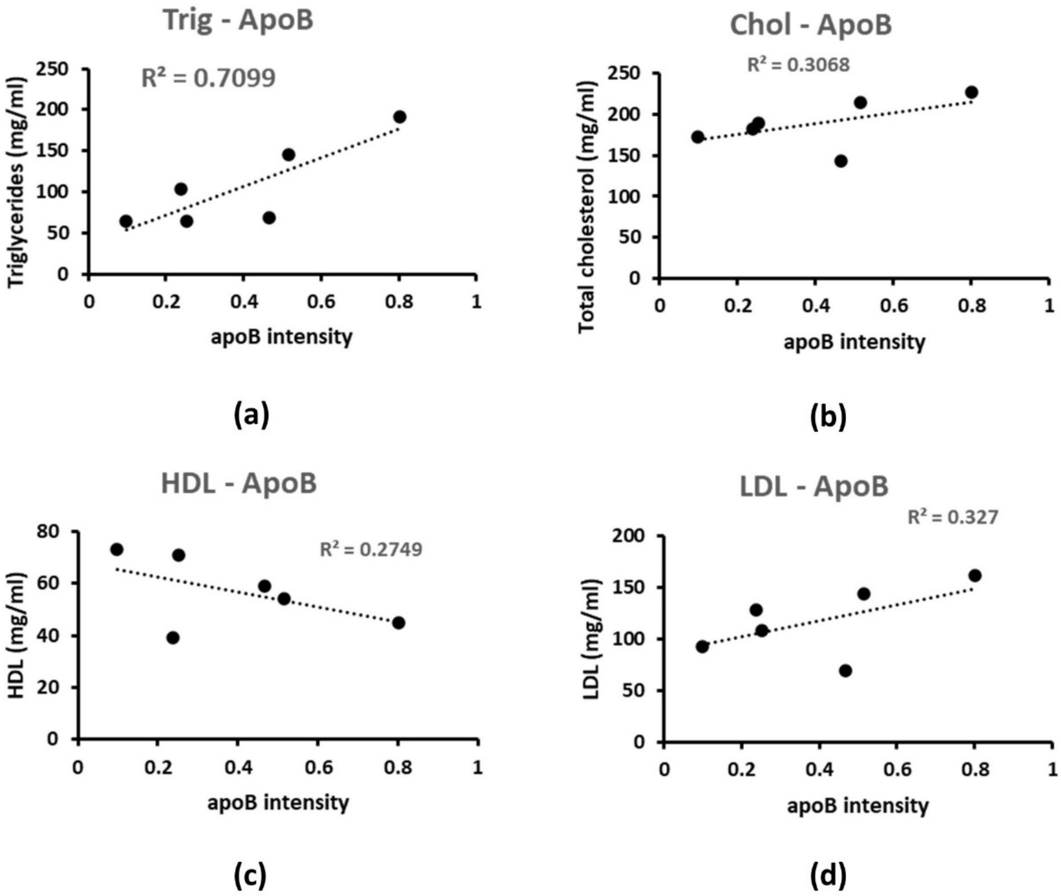

Supplementary Figure S4. The correlation between donor lipid profiles (Triglycerides, total cholesterol, HDL, LDL) measured in serum and ApoB intensity measured by ELISA of preparations of small extracellular vesicle (sEV) isolated by Exospin™. Each dot corresponds to one individual. ApoB mean intensity is an average of 3 replicates. The graph (**a**) shows the correlation between triglycerides concentrations in serum and ApoB intensity in corresponding sEV preparations. The graph (**b**) shows the correlation between total cholesterol concentrations in serum and ApoB intensity in corresponding sEV preparations. The graph (**c**) shows the correlation between HDL concentrations in serum and ApoB intensity in corresponding sEV preparations. The graph (**d**) shows the correlation between LDL concentrations in serum and ApoB intensity in corresponding sEV preparations. Abbreviations: HDL - high-density lipoprotein; LDL - low-density lipoprotein; ELISA - enzyme-linked immunoassay.

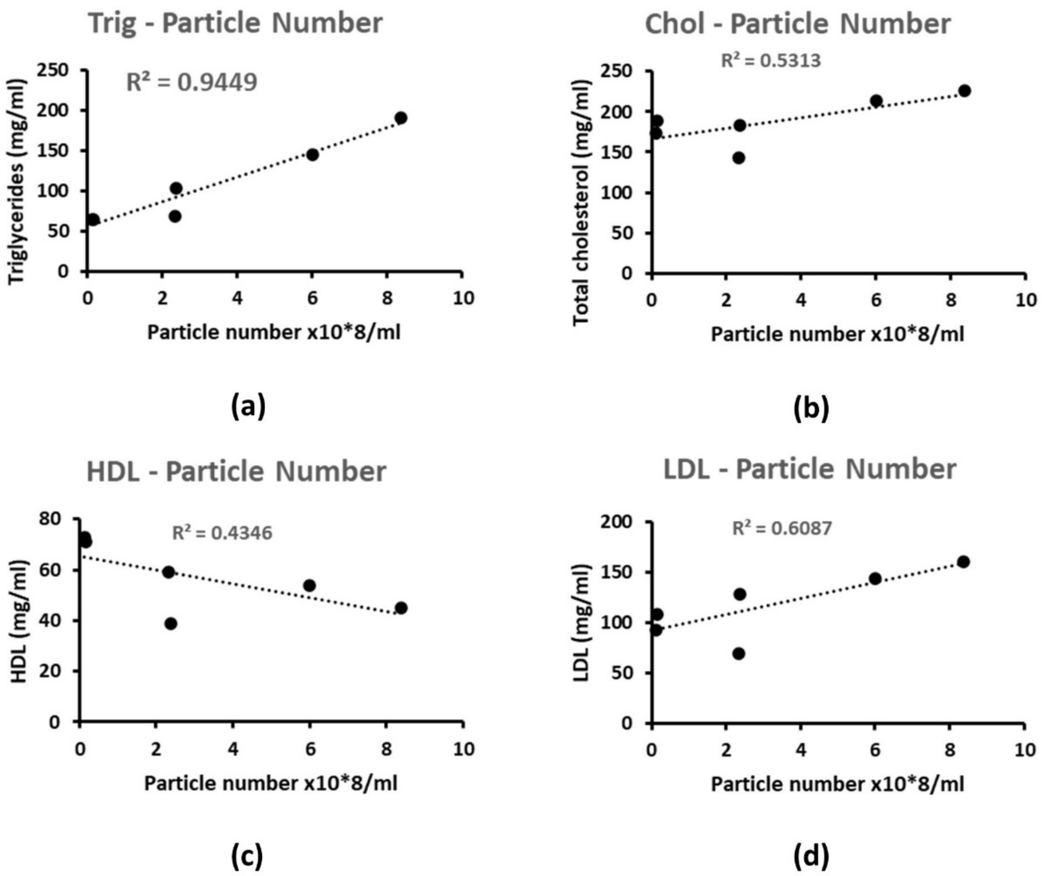

Supplementary Figure S5. The correlation between donor lipid profiles (Triglycerides, total cholesterol, HDL, LDL) measured in serum and the particle number of preparations of small extracellular vesicle isolated by Exo-spin™. Each dot corresponds to one donor. Particle number is an average of 3 nanoparticle tracking analysis measures. The graph (**a**) shows the correlation between triglycerides concentrations in serum and particle numbers in corresponding sEV preparations. The graph (**b**) shows the correlation between total cholesterol concentrations in serum and particle numbers in corresponding sEV preparations. The graph (**c**) shows the correlation between HDL concentrations in serum and particle numbers in corresponding sEV preparations. The graph (**d**) shows the correlation between LDL concentrations in serum and particle numbers in corresponding sEV preparations. Abbreviations: HDL—high-density lipoprotein; LDL—low-density lipoprotein.

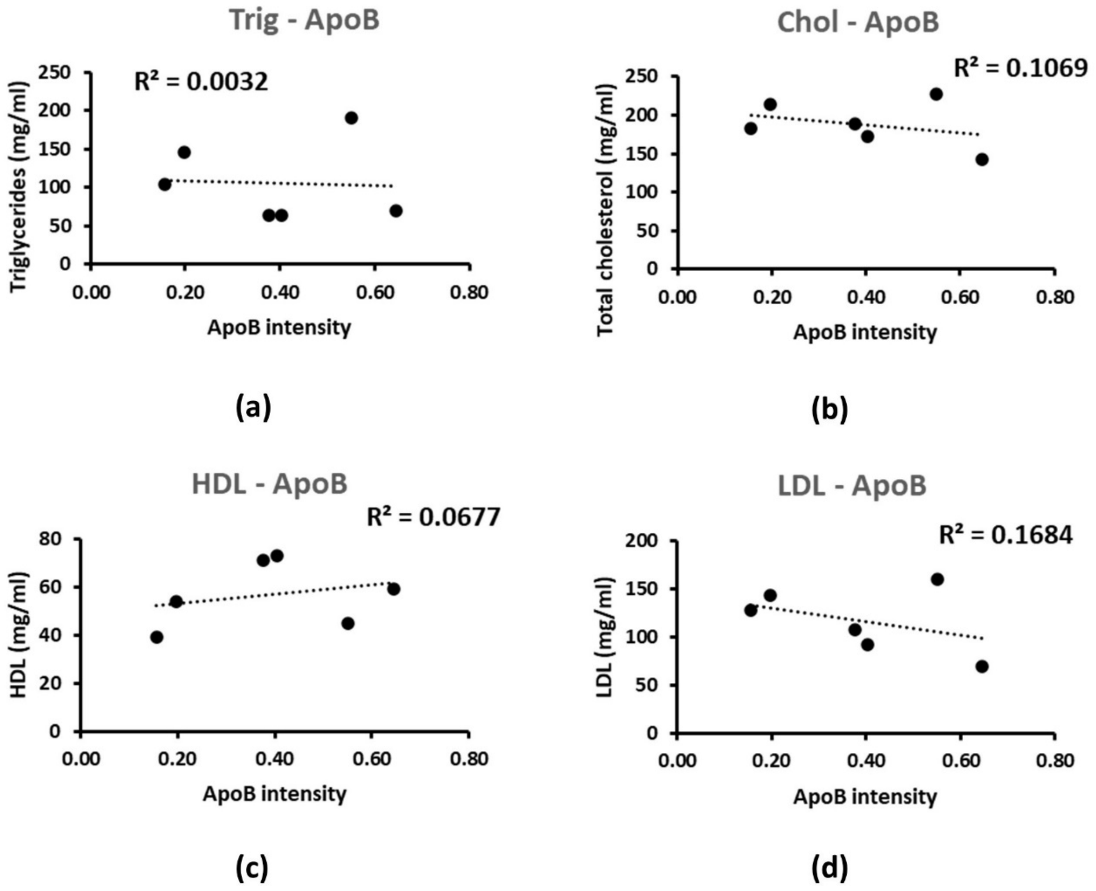

Supplementary Figure S6. The correlation between donor lipid profiles (Triglycerides, total cholesterol, HDL, LDL) measured in serum and ApoB intensity measured by ELISA of preparations of small extracellular vesicle isolated by differential ultracentrifugation. Each dot corresponds to one individual. ApoB mean intensity is an average of 3 replicates. The graph (**a**) shows the correlation between triglycerides concentrations in serum and ApoB intensity in corresponding sEV preparations. The graph (**b**) shows the correlation between total cholesterol concentrations in serum and ApoB intensity in corresponding sEV preparations. The graph (**c**) shows the correlation between HDL concentrations in serum and ApoB intensity in corresponding sEV preparations. The graph (**d**) shows the correlation between LDL concentrations in serum and ApoB intensity in corresponding sEV preparations. Abbreviations: HDL—high-density lipoprotein; LDL—low-density lipoprotein; ELISA—enzyme-linked immunoassay.

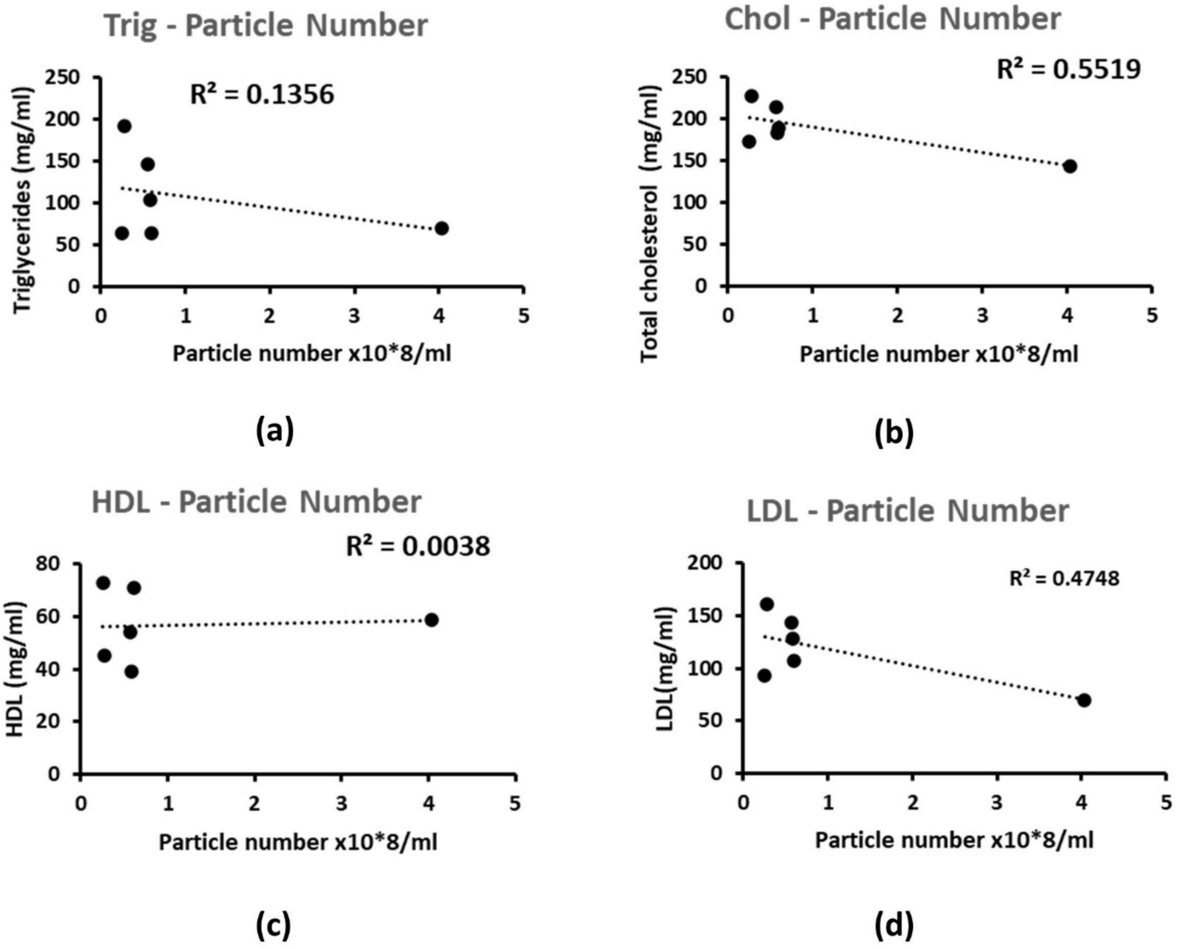

Supplementary Figure S7. The correlation between donor lipid profiles (Triglycerides, total cholesterol, HDL, LDL) measured in serum and the particle number of preparations of small extracellular vesicle isolated by differential ultracentrifugation. Each dot corresponds to one donor. Particle number is an average of 3 nanoparticle tracking analysis measures. The graph (**a**) shows the correlation between triglycerides concentrations in serum and particle numbers in corresponding sEV preparations. The graph (**b**) shows the correlation between total cholesterol concentrations in serum and particle numbers in corresponding sEV preparations. The graph (**c**) shows the correlation between HDL concentrations in serum and particle numbers in corresponding sEV preparations. The graph (**d**) shows the correlation between LDL concentrations in serum and particle numbers in corresponding sEV preparations Abbreviations: HDL—high-density lipoprotein; LDL—low-density lipoprotein.

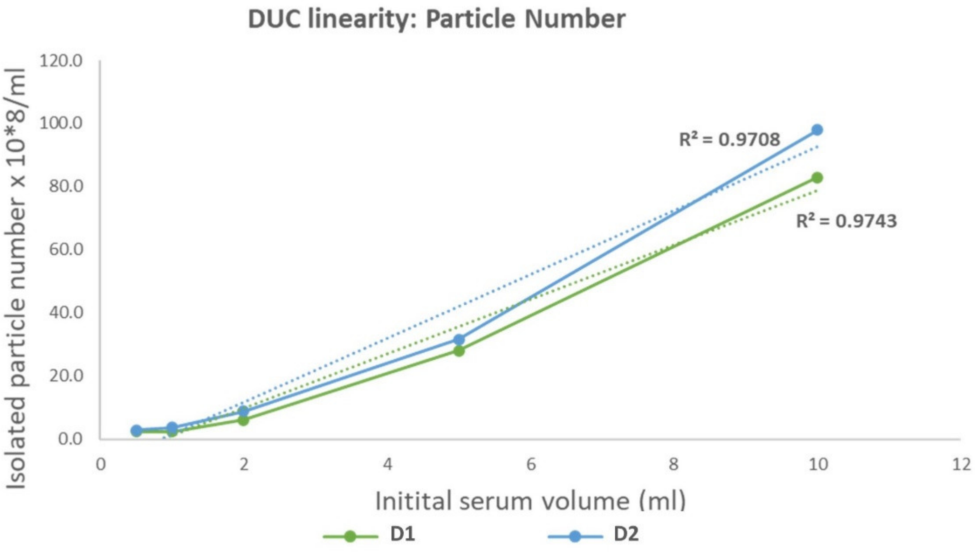

(a)

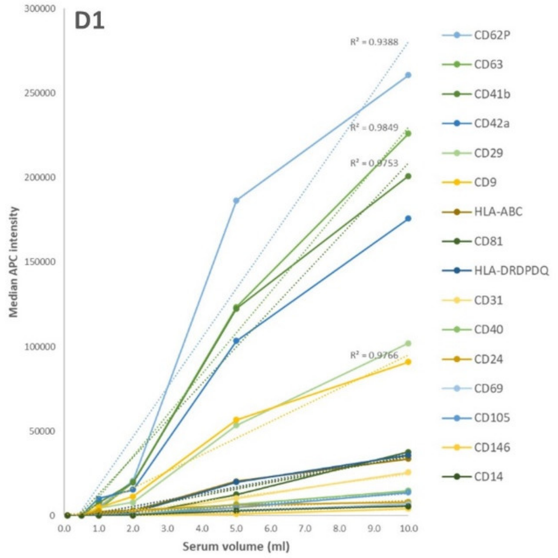

(b)

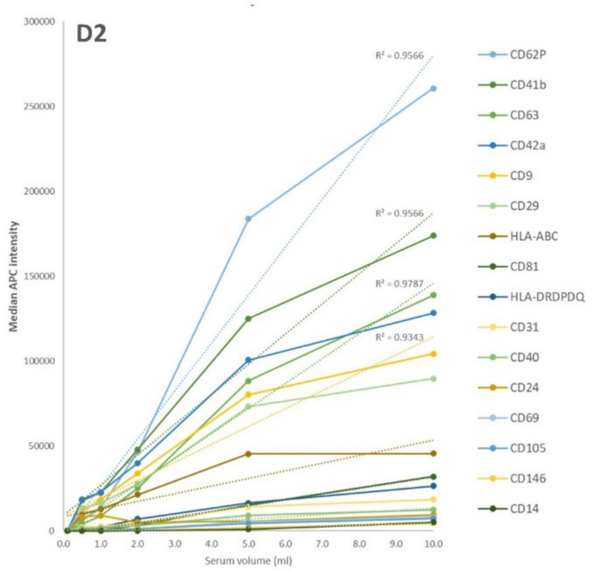

(c)

Supplementary Figure S8. Linearity of differential ultracentrifugation. Graph (**a**) shows the correlation between isolated particle number counted by NTA and the applied initial serum volume for 2 individuals (D1 and D2). Graphs (**b**) and (**c**), respectively donor 1 (D1) and donor 2 (D2) present the median APC signals acquired by MACSPlex exosome human kit that is equivalent to expression of specific surface proteins shown in different colour. Markers are correlated to the applied initial serum volume (0,5 mL; 1 mL; 2 mL; 5 mL; 10 mL). Abbreviations: NTA—nanoparticle tracking analysis. Based on this data, the linearity of differential ultracentrifugation is estimated for 1 to 5 mL of serum.

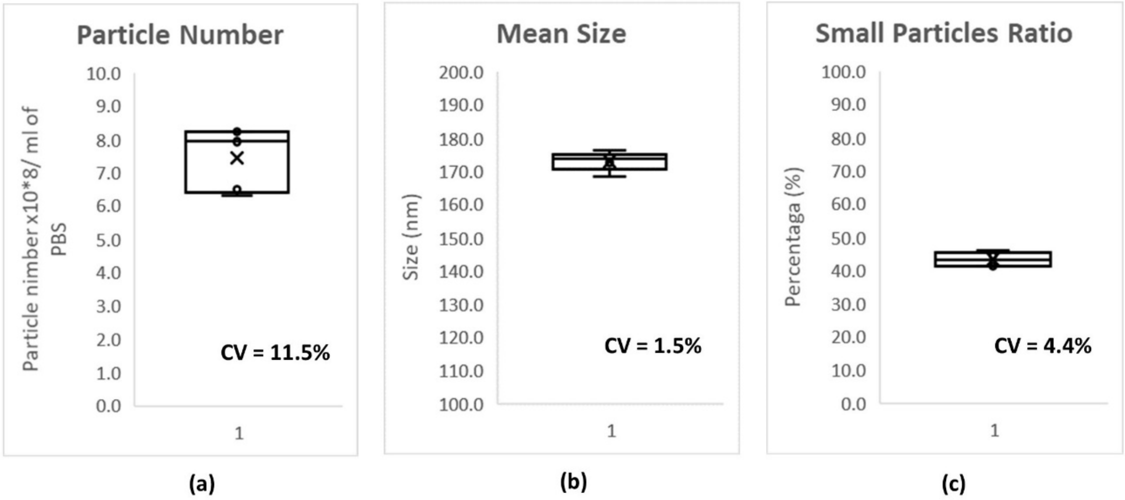

Supplementary Figure S 9. Repeatability of differential ultracentrifugation. Small extracellular vesicles were isolated from the same serum sample in volume of 2 ml each ($n = 5$). Graph (**a**) shows average purified particle number. Graph (**b**) shows particles size and its mean of diameter. Graphs (**c**) shows the percentage of particles that are smaller than 150 nm in diameter. Each dot corresponds to a single sample that is the mean of 3 nanoparticle tracking analysis replicates. CV = Coefficient of variation.
